# Factors affecting time of access of in-patient care at Webuye District hospital, Kenya

**DOI:** 10.4102/phcfm.v8i1.898

**Published:** 2016-10-14

**Authors:** Maxwell M. Lodenyo, Barasa K. Otsyula, Raymond Downing, Kenneth Yakubu, Miriam Miima, Okoye Ifeyinwa

**Affiliations:** 1 Department of Family Medicine, School of Medicine, Moi University, Kenya; 2 School of Medicine, Kenya Methodist University, Kenya; 3 Department of Family Medicine, University of Jos, Nigeria; 4 Jos University Teaching Hospital, Jos Plateau State, Nigeria; 5 Aga Khan Hospital, Dar-es-Salaam, Tanzania; 6 Department of Family Medicine, University of Calabar Teaching Hospital, Cross River, Nigeria

## Abstract

**Background:**

Among many Kenyan rural communities, access to in-patient healthcare services is seriously constrained. It is important to understand who has ready access to the facilities and services offered and what factors prevent those who do not from doing so.

**Aim:**

To identify factors affecting time of access of in-patient healthcare services at a rural district hospital in Kenya.

**Setting:**

Webuye District hospital in Western Kenya.

**Methods:**

A cross-sectional, comparative, hospital-based survey among 398 in-patients using an interviewer-administered questionnaire. Results were analysed using SPSS V.12.01.

**Results:**

The median age of the respondents, majority of whom were female respondents (55%), was 24 years. Median time of presentation to the hospital after onset of illness was 12.5 days. Two hundred and forty seven patients (62%) presented to the hospital within 2 weeks of onset of illness, while 151 (38%) presented after 2 weeks or more. Ten-year increase in age, perception of a supernatural cause of illness, having an illness that was considered bearable and belief in the effectiveness of treatment offered in-hospital were significant predictors for waiting more than 2 weeks to present at the hospital.

**Conclusion:**

Ten-year increment in age, perception of a supernatural cause of illness (predisposing factors), having an illness that is considered bearable and belief in the effectiveness of treatment offered in hospital (need factors) affect time of access of in-patient healthcare services in the community served by Webuye District hospital and should inform interventions geared towards improving access.

## Introduction

Access to health care refers to the timely use of health services to achieve the best possible outcomes,^1^ or the ease with which an individual can obtain needed medical services.^2^ A broader definition identifies the following dimensions of access: availability (distances and travel mode to facilities), acceptability (reasons for provider choice, including reasons for delayed care) and affordability.^3^

Utilisation of healthcare services is an important determinant of health and has particular relevance as a public health and development issue in low-income countries.^4,5,6,7^ In fact, utilisation of healthcare services for the most vulnerable and underprivileged populations has been recommended by the World Health Organization as a basic primary healthcare concept.^8^ Healthcare should ideally be universally accessible without barriers based on affordability, physical accessibility or acceptability of services.^7,9^

In many parts of sub-Saharan Africa, healthcare services remain out of reach for significant proportions of the populace. More than 50% of the populace does not have access to modern healthcare facilities.^10^ Many African national health systems are under stress because of weakening human resource capacity and financing constraints.^11^ The public sector, which is the main provider of healthcare services in most rural areas of Africa, is beset by problems such as lack of equipment and shortages of essential consumables.^12^ Provision of emergency and essential healthcare interventions that are simple but vital is hindered by lack of adequate personnel.^13^ Alarmingly, in many countries functioning hospital services simply do not exist or cannot be reached.^14^ Cultural and educational factors may obscure recognition of illness and benefits from health care.^15^

As a result, many patients with health problems that are routinely treatable in high-income countries never reach a health facility, present late or are treated in a facility with inadequate human or physical resources.^16^ Many deaths still occur for reasons that could have been avoided by provision of simple appropriate care at the right time.^17^

Driven by a health agenda that supports the UN Millennium Development Goals, efforts are underway across Africa to improve access to health care and reduce barriers to service uptake.^18,19^ Although several health initiatives have improved delivery of selected health interventions, other priority interventions still have unacceptably low coverage.^20,21,22,23^

Modern study of healthcare use and access has shifted from an individual-level focus to a combination of the individual, the healthcare system, the external environment and the effects that each has on the others.

The Andersen Model, which was applied in this study, lists three groups of determinants of healthcare access and utilisation: predisposing, enabling and need factors.^24^ Predisposing factors are the sociocultural characteristics of individuals that exist prior to their illness and include demographic factors, health beliefs and social structure. Enabling factors encompass the logistical aspects of obtaining care and include means and know-how to access healthcare services, income, health insurance, a regular source of care, travel, extent and quality of social relationships, available health facilities and personnel and waiting time. Need factors are the most immediate cause of health service use resulting from health problems that generate the need for healthcare services.

Among many Kenyan rural communities, availability of health services is seriously constrained and provision of essential care is limited. Even with the increase in health work force and the number of hospital beds, in-patient health service inequalities persist.^25^

Key health impact indicators, for example maternal and under-5 mortality ratios, suggest a decline or stagnation in health status. While this stagnation may be attributable to the high disease burden because of existing and new conditions as well as inadequate response in addressing the disease burden, the health impact indicators also suggest wide disparities in patients’ access to in-patient healthcare services across the country.^25,26^

Previous studies carried out in Kenya on access to health care have emphasised on income and healthcare-associated costs as the main barriers to the use of healthcare services.^27^ However, there is a dearth of evidence on how other factors influence timely use of in-patient healthcare services. Furthermore, reliable routine data do not exist in Kenya on which to base strategies and targets for reducing inequalities in timely access to in-patient healthcare services.

In order to bring about improvements in health in the communities served by the Webuye District hospital, where cases of late presentation are still rampant, it is essential to understand the demographics of those currently using the facilities that are available, at what time after the onset of illness they seek care as well as ascertain determinants of timely access to in-patient care. Information acquired from this study will be useful in similar practice settings in Africa.

### Research question

What factors influence time of access of in-patient healthcare services at the Webuye District hospital?

### Aim

To identify factors affecting time of access of in-patient healthcare services at a rural district hospital in Kenya.

### Specific objectives

- Establish the first health seeking actions taken by patients on falling sick.
- Determine factors associated with the use of in-patient healthcare services at Webuye District hospital.
- Determine the relationship between these factors and the time of presentation at Webuye District hospital.

## Research methods and design

### Study design

This was a cross-sectional, comparative, hospital-based survey.

### Study setting

Webuye District hospital is located along the Eldoret-Malaba Highway in Bungoma County, Western Kenya. It is a 226-bed hospital, with approximately 150% bed occupancy rates and a catchment population of close to 250,000 persons.^28^ It is also a referral institution for surrounding districts.

### Study population

Patients admitted to the wards were enrolled in the study. The minimum sample size was calculated using the Taro Yamane formula^29^ for sample size determination for estimating proportion in a finite population.

$$\text{sample size }n=\frac{N}{1+N{\left(e\right)}^{2}}$$ [Eqn 1]

Where *n* = Minimum required sample size.

*N* = Finite population – Yearly average of ten thousand in-patients.^28^

*e* = Level of precision – 0.05 with a 95% confidence interval (CI).

$$n=10,000=\frac{385}{1+10,000{\left(0.05\right)}^{2}}$$ [Eqn 2]

Consecutive sampling technique was used where every admitted patient meeting the inclusion criteria and consenting to participate in the study was enrolled. This was carried out until the sample size was achieved. Consecutive sampling was justified by the absence of a sampling frame and the fact that these patients did not have unique identities or characteristics.

### Inclusion criteria

- Patients admitted for management in the wards.

### Exclusion criteria

- Readmissions previously enrolled in the study.
- Unaccompanied critically ill and mentally unstable patients, owing to ethical considerations.

### Data collection

The data collection tool was a self-designed questionnaire (Appendix 1) based on the conceptual framework of the Andersen Model. Input of physicians who were knowledgeable and experienced in hospital systems management was sought to ascertain the content validity of the questionnaire. Prior to commencement of the main study, a pilot study involving 40 patients was carried out among in-patients to identify areas of ambiguity in language used, ascertain ease of administration and confirm accuracy of the study methodology. Questions touching on health insurance and income (enabling factors) were omitted from the questionnaire owing to difficulty in eliciting these responses and the fact that most respondents did not have insurance or a regular income. Four research assistants were recruited by the Principal Investigator and trained on the objectives of the study, administration of the questionnaire and obtaining informed consent.

The questionnaire was interviewer-administered to enrolled respondents during their hospital stay. The research assistants explained all the questions to the respondents and filled in the questionnaires during the interviews. Data were collected between 1 March and 30 April 2009. Monitoring and supervision of the research process and its progress was undertaken by the Principal Investigator to ensure adherence to research protocol and quality. Data were checked for completeness and coded by the Principal Investigator. Entry was performed in EpiData and later exported to SPSS V.12.01 for statistical analysis.

### Data analysis

The independent variables of interest were age, gender, marital status, level of education, occupation, perception on the cause of illness (all predisposing factors); time to hospital, cost of transportation to hospital, means of transportation, concerns when coming to hospital (all enabling factors); decision to come to hospital and reasons for coming to hospital (need factors). The primary outcome measure was the duration of illness at the time of presentation to the hospital.

Frequency tables and measures of central tendency [median and interquartile range (IQR)] were generated for the demographic characteristics (i.e. marital status, education, occupation) of the patients or guardians of the patients when minors were involved.

The median and interquartile range were used as measures of central tendency and spread, respectively, instead of the mean owing to the nonparametric distribution of the data and the presence of extreme values (outliers) to which the mean is extremely sensitive.

Chi-square test was used to test for association between the categorical variables (i.e. gender, marital status, level of education, occupation, means of transportation, decision to go to the hospital, perception on cause of illness, concerns when coming to hospital, reason for coming to hospital) and grouped time between onset of illness and presentation at the hospital. Mann–Whitney–Wilcoxon rank sum test was used to test for association between medians of continuous variables (i.e. age, time to hospital, etc.) and time between onset of illness and presentation at the hospital. Controlling for confounders, adjusted odds ratios by binomial logistic regression was used to identify factors significantly associated with duration of illness at the time of presentation to the hospital. A *p*-value of less than 0.05 was considered statistically significant.

## Ethical considerations

Written, informed consent was sought from all respondents who included guardians of minors and patients who were unable to give consent. Respondents were assured that all information obtained was to be treated in the strictest confidence and was to be used for study purposes only. Access to the data obtained was limited to the interviewer and Principal Investigator. Respondents had the right to refuse participation and withdraw at any stage of the interview. No patient was denied treatment for declining to participate in the study or had treatment delayed because of the interview process. No inducements, financial or otherwise, were offered to encourage participation. Approval was sought and obtained from the Institutional Research and Ethics Committee of Moi University (approval number 000336, irec number, irec/2008/36) and Webuye District hospital administration before commencement of the study.

## Results

In total, 398 respondents were enrolled, of which 115 were minors (17 years of age and below) who were brought to the hospital by their guardians. Of the 115 minors, 85 (84%) were children aged below 5 years. The median age (IQR) of all the respondents was 24 (11.5, 35.3); the youngest patient was a new-born while the oldest was 85 years old. Two hundred and nineteen (55%) patients were female patients and 225 (56.5%) respondents were self-employed. Majority of the patients or their parents/guardians were married (71.4%) and close to half of the respondents- 198 (49.7%)- had primary school education. The median duration (IQR) from patient’s home to hospital, amount spent on transportation to the hospital, as well as other baseline details, are shown in Table 1.

**TABLE 1:** Descriptive characteristics of study population (*n* = 398).

Characteristics	Median (interquartile range)
Time to hospital (hours)	1 (0.5, 1.5)
Amount spent to hospital (shillings)	100 (50.200)†
Age (years)	24 (11.5, 35.3)
**Grouped age (years) *n* (%)**	
0–20	172 (43.2)
21–40	156 (39.2)
41–60	43 (10.8)
61–80	24 (6.0)
≥ 81	3 (0.8)
**Gender**	
Male	179 (45)
Female	219 (55)
**Marital status**	
Single	89 (22.4)
Married	284 (71.4)
Other (divorced, separated, widowed)	25 (6.2)
**Education level**	
None	39 (9.8)
Primary	198 (49.7)
Secondary	140 (35.2)
Tertiary	21 (5.3)
**Occupation**	
Formal	35 (8.8)
Self-employed	225 (56.5)
Unemployed	91 (22.9)
Student	47 (11.8)

*Source*: Own primary research results

† equivalent to one US dollar.

Among the paediatric age group (12 years of age and below), the most common presenting diagnoses were malaria, acute respiratory tract infections, acute diarrhoeal diseases, malnutrition and anaemia.

Among the patients admitted to the adult wards, the most common admitting diagnoses were malaria, acute infections (soft tissue, bone, respiratory, urinary tract and gynaecological), acute pregnancy complications, injuries, malignancies and acute surgical emergencies.

At the time of presentation for in-patient care, a total of 247 patients (62%) had been sick for less than 2 weeks, while 73 (18.3%) had been sick for more than 2 weeks but less than 1 month. A total of 78 patients (19.7%) had been sick for periods ranging from several months to years (Table 2). Median time of presentation (IQR) to the hospital after onset of illness was 12.5 days (6.2, 24.3).

**TABLE 2:** Duration of illness as at presentation among adult and paediatric patients.

Duration	Paediatric patients *n* (%)	Adult patients *n* (%)	Total *n* (%)
< 2 weeks	94 (93.1)	153 (51.5)	247 (62)
> 2 weeks, < 4 weeks	4 (3.96)	69 (23.2)	73 (18.3)
Several months	3 (2.94)	49 (16.5)	52 (13.1)
Years	0	26 (8.8)	26 (6.6)
**Total**	**101**	**297**	**398**

*Source*: Own primary research results

Three hundred and twenty nine of the respondents (82.7%) came to the hospital by vehicle or motorcycle, 42 (10.5%) on bicycle, 16 (4%) combined bicycle with motorcycle or vehicle, while 11 (2.8%) came on foot.

In response to what they perceived to be the factor that would ‘most’ affect their use of healthcare services generally, 143 of the respondents (36%) cited cost as the biggest barrier to access of in-patient services. Other barriers as perceived by patients/guardians prior to admission at the hospital are shown in Table 3.

**TABLE 3:** Distribution of respondents by perceived barriers to the use of in-patient health services.

Barrier (concerns)	*n* (%)
Cost	143 (36)
Non-availability of medical staff	111 (28)
Distance to facility	104 (26)
Time of day	36 (9)
Non-availability of drugs	4 (1)
**Total**	**398**

*Source*: Own primary research results

Majority of the respondents- 302 (75.9%)- perceived their illness as ‘primarily’ attributable to natural causes, while 51 (12.8%) believed their illness was as a result of a supernatural phenomenon, for example a curse or witchcraft. Forty-five respondents (11.3%) believed their illness was because of an accident.

Most respondents- 262 (65.8%)- reported that the decision to come to hospital was made by others. In six out of 10 cases, male relatives made this decision. One hundred and thirty six (34.2%) made the decision independently.

Slightly more than half of the patients- 203 (51%)- came to the hospital because the illness was unbearable, more than one-third (35%) because they believed they would be treated effectively, while 24 (6%) reported that herbs hitherto used were ineffective as shown in Table 4.

**TABLE 4:** Distribution of respondents by their reasons for coming to the hospital.

Reason	*n* (%)
Illness unbearable	203 (51)
Believed would be effectively treated	139 (35)
Obtained some money	28 (7)
Herbs ineffective	24 (6)
Assisted by relatives	4 (1)
**Total**	**398**

*Source*: Own primary research results

**FIGURE 1:**
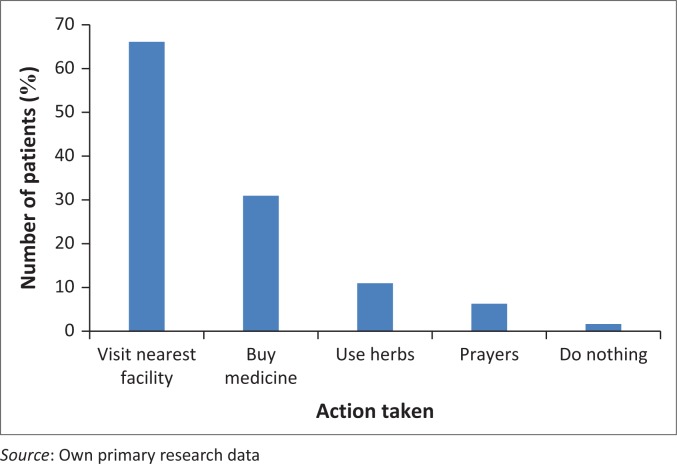
First action taken on falling sick (*n* = 398).

Concerning the ‘first’ action taken on falling sick, more than half- 231 (58%)- of the patients visited the nearest health facility (including the out-patient department of Webuye District hospital, health centres, dispensaries, other district hospitals, and privately owned facilities), 105 (26.4%) bought medicines from the nearest pharmacy while 37 (9.3%) preferred the use of herbs. Twenty one (5.3%) sought relief through prayers (Figure 1).

For purposes of statistical analysis and taking into consideration median time of presentation (12.5 days), respondents were grouped into two categories:

- Those sick for less than or equal to 2 weeks (247 patients).
- Those sick for more than 2 weeks, several months or years (groups collated to give a total of 151 patients).

Statistically significant differences were noted between the two groups with regard to:

- **Predisposing factors**: Age and perception of the cause of illness (*p* < 0.05). Gender, marital status, education and occupation were not significant.
- **Enabling factors**: Time taken to reach the hospital and the amount of money spent to get to the hospital (*p* < 0.05). Concerns when coming to hospital and means of transport were not statistically significant.
- **Need factors**: Decision to go to the hospital and the reason for coming to the hospital (*p* < 0.05). (Table 5a and Table 5b).

**TABLE 5a:** Association between predisposing, enabling, need factors and grouped duration of illness as at presentation.

Independent variable	Duration of illness as at presentation		Chi-square / *z*-value	*p*-value
	< 2 weeks	> 2 weeks		
*Median age in years (IQR)	20 (3, 30)	32 (20, 52)	7.185	< 0.001
*Median time to hospital in hours (IQR)	1 (0.5, 1)	1 (1, 2)	4.373	< 0.001
*Median amount spent to hospital in shillings (IQR)	100 (50, 180)	150 (100, 210)	5.711	< 0.001

**TABLE 5b:** Association between predisposing, enabling, need factors and grouped duration of illness as at presentation.

Independent variable	Duration of illness as at presentation		Chi-square /*z*-value	*p*-value
	< 2 weeks *n* = 247 (%)	> 2 weeks *n* = 151 (%)		
**Gender**				
Female	142 (57.5)	77 (51)	1.598	0.214
Male	105 (42.5)	74 (49)	-	-
**Means of transport**				
Vehicular	196 (79.3)	133 (88)	3.087	0.90
Other	51 (20.7)	18 (12)	-	-
**Marital status**				
Married	180 (72.9)	104 (68.9)	-	-
Single	57 (23.1)	32 (21.1)	5.526	0.063
Others (divorced, separated, widowed)	10 (4)	15 (10)	-	-
**Educational level**				
None	17 (6.9)	22 (14.6)	-	-
Primary	131 (53)	67 (44.4)	7.281	0.063
Secondary	87 (35.2)	53 (35.1)	-	-
Tertiary	12 (4.9)	9 (5.9)	-	-
**Occupation**				
Formal	22 (8.9)	13 (8.6)	-	-
Unemployed	54 (21.9)	37 (24.5)	0.403	0.940
Self-employed	142 (57.5)	83 (55)	-	-
Student	29 (11.7)	18 (11.9)	-	-
**Decision to go to hospital**				
Self	101 (40.9)	35 (23.2)	-	-
Referred	1 (0.4)	0	13.069	0.001
Others	145 (58.7)	116 (76.8)	-	-
**Perceived cause of illness**				
Accident	26 (10.5)	19 (12.6)	-	-
Supernatural	15 (6.1)	36 (23.8)	28.292	< 0.001
Natural causes	206 (83.4)	96 (63.6)	-	-
**Concerns when coming to hospital**				
Hospital issues	2 (0.8)	0	-	-
Time of day	28 (11.3)	8 (5.3)	5.088	0.064
Cost/distance to hospital	217 (87.9)	143 (94.7)	-	-
**Reason for coming to hospital**				
Illness unbearable	140 (56.7)	62 (41.1)	-	-
To be treated effectively	87 (35.2)	51 (33.8)	23.296	< 0.001
Others	20 (8.1)	38 (25.1)	-	-

*Source*: Own primary research results

IQR, interquartile range.

*, Compared using Mann–Whitney test, others compared using Chi-square test (*p*< 0.05).

Adjusted odds ratios by binomial logistic regression were used to compare the odds of seeking treatment within and after 2 weeks of falling ill with reference to age, amount spent and time taken en route the hospital, reasons for coming to the hospital, who made the decision to come to the hospital and perception about the cause of illness.

Adjusting for all other factors, predisposing factors (10-year increment in age and perception of a supernatural cause of illness) and need factors (having an illness that was considered bearable and belief in effective treatment at the hospital) were significant predictors of the time of presentation to the hospital (Table 6).

**TABLE 6:** Binomial logistic regression (unadjusted and adjusted odds ratios for factors associated with time of presentation).

Factor	Unadjusted OR (95% CI)	Adjusted OR (95% CI)
Time to hospital	1.008 (1.003–1.013)	1.002 (0.996–1.007)
Amount spent to hospital	1.000 (1.000–1.001)	1.000 (1.000–1.001)
Age per 10 years increase	1.600 (1.300–1.800)	1.600 (1.480–1.970)
**Reason for coming to hospital (ref = other)**		
Illness unbearable	0.233 (0.126–0.433)	0.237 (0.107–0.523)
To be treated effectively	0.309 (0.162–0.587)	0.375 (0.167–0.842)
**Who prompted to come to hospital (ref = self)**		
Others	2.293 (1.454–3.615)	1.669 (0.978–2.847)
**Perception on cause of illness (ref = accident)**		
Curse/witchcraft	3.284 (1.412–7.640)	3.247 (1.192–8.842)
Natural	0.638 (0.337–1.208)	1.135 (0.544–2.367)

*Source*: Own primary research results

CI, confidence intervals; OR, odds ratio.

The odds of presenting with illness lasting longer than 2 weeks increased 1.6 times for every 10-year increase in age (OR: 1.6, 95% CI: 1.480–1.970).

The odds of presenting later than 2 weeks after the onset of the illness was three times higher for respondents who attributed their illness to supernatural causes compared to those who did not (OR: 3.247, 95% CI: 1.192–8.842).

The odds of presenting later than 2 weeks after the onset of illness was about four times lower among patients whose illness was unbearable compared to those whose illness was bearable (OR: 0.237, 95% CI: 0.107–0.523).

Those who believed that they would be treated effectively at the hospital were also less likely to present to hospital after 2 weeks (OR: 0.375, 95% CI: 0.167–0.842).

## Discussion

We found that incremental increase in age, perception of a supernatural cause of illness (predisposing), having an illness that was considered bearable and belief in effective treatment in hospital (need) were the factors most likely to predict time of presentation for in-patient care at the hospital.

In this study, 55% (219/398) of the respondents were female respondents, compared to 45% who were male respondents. This is in keeping with findings by Redondo-Sendino *et al*.^30^ who reported that compared to men, women report greater morbidity and make greater use of healthcare services. Close to two-thirds of the respondents (71.4%) were married. While not a significant predictor of utilisation of in-patient services in this study, other studies^31,32^ have shown advantages of being married on the health status of the individual. Slightly below half of the respondents had primary school level education, while a quarter were unemployed, comparable to findings by Harris and others in South Africa.^33^

With every 10-year increase in age, the odds that the patient would present to the hospital having been ill for more than 2 weeks increased 1.6 times. The younger the patient, the more likely they would present or be brought to the hospital sooner after onset of the illness. This finding is in agreement with studies by Tao^34^ and Young.^35^ In contrast, similar studies on access to healthcare services demonstrated that rates of health service utilisation increased with advancing age.^36,37^ Delayed access to in-patient hospital services by older patients may be attributable to reduced mobility, concerns about privacy, use of alternative medicine, lack of support from family members with a possible preference to reserve scarce resources for younger members of the family.

Previous studies have observed that perception of illness may be associated with avoidance of healthcare facilities or significant delay in seeking effective treatment.^38^ Approximately one in every four respondents (23.8%) of the 151 study participants who presented with sickness lasting longer than 2 weeks attributed their illness to supernatural causes. The odds of these patients presenting later than 2 weeks to the hospital was three times more than those who attributed their illness to natural causes or accidents. Similar findings on perceptions of illness and health seeking behaviour were reported in studies conducted in Burkina Faso and South Africa.^38,39^

In this study, patients who found their illness unbearable or believed they could be treated effectively were less likely to present to the hospital later than 2 weeks after onset of the illness. Severity of the illness experience influenced use of in-patient hospital services. These findings are in agreement with those of Chean *et al*.^40^

Concerning the ‘first’ action on falling ill, 58% of the patients visited the nearest health facility. This may be explained by proximity of the facilities to their homes or by the success of sensitization efforts by health extension workers and local administrative authorities on the need for timely presentation at a health facility when ill. Approximately 26% of the respondents in this study reported that they would buy medication from the nearest pharmacy, while 9.3% resorted to traditional herbs. This is in contrast with similar studies in Ethiopia, Malawi and Somalia, which demonstrated that rural populations tend to follow a pattern of alternative health practices in response to illness.^41,42,43^

The findings of this study are in contrast with those studies in rural Greece, which demonstrated a comparatively higher rate of self-medication at 76%^44^ with lower rates in Spain and Denmark at 11% and 3%, respectively.^45,46^ The comparatively lower rate of self-medication in this study may be because of neglect of the initially mild nature of illness, high cost of medication from private pharmacies and proximity of healthcare facilities offering medication at subsidised rates or, as stated above, the success of community outreach projects.

In this study, the decision to bring a patient to the hospital was in two out of three cases made by someone other than the patient or guardian and in most cases - 6 out of 10 cases - by male relatives. Seventy seven percent of the 151 patients who presented with illness that had lasted more than 2 weeks had the decision to come to hospital made by others compared to 59% percent of the 247 patients in the group presenting earlier to hospital. This is in agreement with studies in Burkina Faso, Mali and Nigeria where husbands alone make decisions on access to healthcare services by their spouses and children.^47^

This could be explained by the fact that many of the sick individuals would, before seeking in-patient hospital care, make contact with others with whom they had close ties such as family members, friends, neighbours, workmates and employers. These people would influence the sick individual in the choice of therapy, meet costs and take care of the sick person throughout illness.

Other predisposing factors (i.e. gender, marital status, educational level and occupation and enabling factors, i.e. amount and time spent to hospital as well as means of transport) were not significant predictors of the duration of illness as at the time of presentation to the hospital for in-patient care. This is in contrast with studies conducted in other countries where these factors were found to be significant with regard to the time of access to in-hospital services.^48,49,50,51,52^

Cost, non-availability of medical personnel at health facilities and distance to facilities were perceived as the top barriers to use of in-patient services. However, these were not significant predictors of utilisation of these services. These perceptions may be clouded by past experiences and situations in most rural hospitals in Kenya, which for long periods have had little or no supplies and skeletal staffing. Lack of information about subsidised health care, efforts at staffing and the slow pace of infrastructure development may reinforce these perceptions.

### Limitations

The study may have been limited by social desirability and response biases on the part of the respondents. Convenience sampling used may have introduced selection bias. The cross-sectional nature of the study precludes assessment of seasonal patterns of access to in-patient services. The effect of the type of illness and the first action taken on the time of presentation at the hospital was not accounted for.

### Recommendations

Firstly, introducing home visits by community health workers and a special health insurance for the elderly are possible interventions that may increase access to in-patient hospital services in this age group for those who need it.

Secondly, in the development of health system interventions, the inclusion of efforts geared towards fostering therapeutic alliances between the community, out-patient care facilities and the hospital as well as those aimed at improving local understanding of illness, addressing faulty beliefs and cultural practices (e.g. through cultural competency training for healthcare providers) may increase timely access to in-patient hospital services among those who need it.

## Conclusion

At the Webuye District hospital, older patients were more likely to present to the hospital later than the younger patients. Local perceptions of illness influenced the health seeking behaviour of those who required in-patient hospital services and most of the patients who presented within 2 weeks of onset of their illnesses did so because they found their illness unbearable.

## APPENDIX 1

### QUESTIONNAIRE

Factors affecting time of access of in-patient care at Webuye District hospital, Kenya.

Diagnosis……………………………………………

1. Age………..
2. Sex………..
3. Marital Status…………
4. Occupation………..
5. Education (class)……….
6. Religion…………
7. How long have you been sick?
  - Less than two weeks
  - A few weeks (> 2weeks, < 4 weeks)
  - Several months
  - Several years
8. What means did you use to get to hospital today?
  - Vehicle
  - Bicycle
  - Motorcycle
  - Foot
  - Other: *specify…….*
9. How much time did you use to get to hospital today?………
10. How much money did you spend?………..
11. My biggest concern when coming to hospital was:
  - Costs
  - Distance
  - State of roads
  - Means of transport
  - Time of day
  - Availability of drugs
  - Availability of staff/expertise
  - Other: *specify*……….
12. In my opinion, my illness is a result of:
  - A curse or ‘evil eye’
  - An accident
  - A naturally occurring condition
  - Bad weather
  - Divine punishment
  - Witchcraft
  - Other: *specify*……….
13. When I get sick I:
  - Visit the nearest health facility
  - Use herbs
  - Pray with others
  - Buy drugs from the chemist
  - Do nothing because of lack of money
  - Other: *specify*……….
14. Who prompted you to seek treatment now?
  - Self
  - Relatives
  - Neighbours
  - Village Chief
  - Church
  - Was referred
  - Other: *specify*……….
15. Why did you come to the hospital at this moment?
  - Illness unbearable
  - Herbs ineffective
  - Can be treated effectively
  - Got some money
  - Assisted by relatives
  - Other: *specify*……….

## References

[1] Institute of Medicine, Committee on Monitoring Access to Personal Health Care Services Access to health care in America. Washington, DC: National Academy Press; 1993.

[2] RAND Health; Global Health; Center for Military Health Policy Research. Available from: www.rand.org/topics/health-care-access.html (Accessed: 28 March 2010)

[3] Clark D Dimensions of the concept of access to healthcare. Bull New York Acad Med 1983; 51(2): 5–8. PMC1920319 6340770

[4] Mackenbach J, Looman C, Kunst A, Habbema J, Van der Maas P Post-1950 mortality trends and medical care; gains in life expectancy due to declines in mortality from conditions amenable to medical interventions in the Netherlands. Soc Sci Med 1988; 27: 889–94. 3227384 10.1016/0277-9536(88)90278-x

[5] Bonneaux L, Looman W, Barendregt J, Van der Maas P Regression analysis of recent changes in cardiovascular morbidity and mortality in the Netherlands. BMJ 1997; 314: 789–92. 9080996 10.1136/bmj.314.7083.789 PMC2126212

[6] Bayo A, Albert X, Alfonso L, Cortina P, Corelia D The effectiveness of health systems in influencing avoidable mortality: a study in Valencia, Spain 1975–90. J Epidemiol Community Health 1996; 50: 320–5. 8935465 10.1136/jech.50.3.320 PMC1060290

[7] Obrist B, Iteba N, Lengeler C, Makemba A, Mshana C, Nathan R, et al Access to healthcare in contexts of livelihood insecurity. A framework for analysis and action. PLOS Med 2007; 4: 1584–8. 17958467 10.1371/journal.pmed.0040308 PMC2039761

[8] World Health Organization Declaration of Alma-Ata. International Conference on Primary Healthcare, Alma-Ata, USSR; 1978.

[9] Gunford M, Figuera-Munoz J, Myfanwy M, Hughes D, Gibson B, Beech R, et al What does access to healthcare mean? J Health Serv Res Policy 2002; 7: 186–8. 12171751 10.1258/135581902760082517

[10] Akinola Lanre A model of good health. Health care; 2009 Available from: http://www.Pharmaccess.org/FileLib/2009%20TIA.pg48-50_Healthcare03%20 (2).pdf (Accessed 28 September 2015)

[11] World Bank World development indicators 2006 report. Washington, DC.

[12] Eldryd P, Godfrey R, Mabey D, Gill G Principles of medicine in Africa. 3rd ed Cambridge, Cambridge University Press, 2004, pp. 87–95.

[13] World Health Organization Working together for health. World Health Report. World Health Organisation, Geneva, Switzerland, 2006.

[14] Make every mother and child count WHO world health report. Geneva, Switzerland: WHO; 2005.

[15] O’Donnell Owen Access to healthcare in developing countries: breaking down demand barriers. Cad Saude Publica 2007; 23(12): 2820–34. 18157324 10.1590/s0102-311x2007001200003

[16] Kaseje D Healthcare in Africa: challenges, opportunities and an emerging model for improvement. Woodrow Wilson International Center for Scholars’ Meeting, Washington DC, USA; 2006.

[17] King M, Bewes P, Cairns J, Thornton J, Shepherd J, Stewart J, et al Primary surgery vol.1 Oxford: Oxford University Press; 1993, pp. 1–14.

[18] United Nations The millennium development goals, report 2005. New York: United Nations; 2005

[19] Sachs J, McArthur W The millennium project. A plan for meeting the Millennium Development Goals. Lancet 2005; 365: 347–53. 15664232 10.1016/S0140-6736(05)17791-5

[20] The World Health Organization Maximising Positive Synergies Collaborative Group An assessment of interactions between global health initiatives and country health systems. Lancet 2009; 373: 2137–69. 19541040 10.1016/S0140-6736(09)60919-3

[21] United Nations Childrens’ Fund State of the world’s children 2009. New York: UNICEF; 2009.

[22] World malaria report 2008 Geneva: World Health Organization; 2008.

[23] Madon T, Hofman K, Kupfer L, Glass R Public health. Implementation science. Science 2007; 318: 1728–9. 18079386 10.1126/science.1150009

[24] Andersen R Revisiting the behavioural model and access to medical care; does it matter? J Health Soc Behav 1995; 36: 1–10. 7738325

[25] Kenya facts and figures Publisher: Nairobi, Kenya National Bureau of Statistics; 2008.

[26] World Health Organization Country co-operation strategy briefs (Kenya), World Health Organization, Geneva, Switzerland, 2009.

[27] Xu K, James C, Carrin G, Muchiri S World Health Organization. An empirical model of access to healthcare, healthcare expenditure and impoverishment in Kenya: learning from past reforms and lessons for the future. Discussion Paper Number 3; 2006.

[28] Unpublished Webuye District hospital records; 2009.

[29] Taro Y Statistics: An introductory analysis, 2nd ed. New York: Harper and Row; 1967.

[30] Redondo-Sendino A, Guallar-Castillon P, Banegas J, Rodriguez-Artalejo F Gender differences in the utilisation of healthcare services among the older adult population of Spain. BMC Public Health 2006; 6: 155. 16780576 10.1186/1471-2458-6-155 PMC1525176

[31] Hu R, Goldman N Mortality differentials by marital status: An international comparison. Demography 1990; 27: 233–50. 2332088

[32] Rehman O Excess mortality for the non-married in rural Bangladesh. Int J Epidemiol 1993; 22: 445–56. 8359960 10.1093/ije/22.3.445

[33] Harris B, Goudge J, Ataguba J, McIntyre D, Nxumalo N, Jikwana S, et al Inequities in access to healthcare in South Africa. J Publ Health Pol 2009; 3: 77–85. 10.1057/jphp.2011.35 21730985

[34] Tao G, Anderson L, Zhang P Difference in access to healthcare services in rural America by rural-urban classification system. Academy for Health Serv. Res. Health Policy Meet. 2000; 17.

[35] Young J, Menken J, Williams J, Khan N, Kuhn R Who receives healthcare? Age and sex differentials in adult use of healthcare services in rural Bangladesh. World Health Popul 2006; 8(2): 83–100. 18277104 10.12927/whp.2006.18038

[36] Keene J, Li X Age and gender differences in health services utilization. J Public Health 2005; 7(1): 74–9. 10.1093/pubmed/fdh208 15637107

[37] Booth M, Bernard D, Quine S, Kang M, Usherwood T, Alperstein G, et al Access to healthcare among Australian adolescents: young peoples’ perspectives and their socio-demographic distribution. J Adolesc Health 2004; 34(1): 97–103. 14706412 10.1016/j.jadohealth.2003.06.011

[38] Biersmann C, Sanou A, Wladarsch E, De Alegri M, Konyate B, Muller O Malaria in Burkina Faso; Local illness concepts, patterns of traditional treatment and influence on health seeking behavior. Malar J 2007; 6: 106. 17686147 10.1186/1475-2875-6-106 PMC1971712

[39] Wessels H Folk healers in South Africa – traditions cannot be ignored. J Eur Med Writers Assoc 1992; 15(2): 14.

[40] Chean R, Men R, Meessen B, van Pelt M, van Damme W, Lucas H I wish I had AIDS. Qualitative study on access to healthcare services for patients with diabetes and HIV/AIDS in Cambodia. Health, Culture and Society 2012; 2(1): 22–39.

[41] Cook G, Zumla A Manson’s tropical diseases. 22nd ed Philadelphia, PA: Saunders/Elsevier, 2009, p. 38.

[42] Deressa W Treatment-seeking behavior of febrile illness in an area of seasonal Malaria transmission in rural Ethiopia. Malar J 2007; 6: 49. 17462087 10.1186/1475-2875-6-49 PMC1866240

[43] Mwaura W, Moloney G Somali KAP study on infant and young child feeding and health seeking practices. Field Exchange 2008; 33: 7.

[44] Eystathios S, Panagiotis M, Papazafiropolou A, Gikas A, Matzouranis G, Papafragos I, et al Self-medication with antibiotics in a rural population in Greece: a cross-sectional Multi-center study. BMC Fam Pract 2010; 11: 58. 20691111 10.1186/1471-2296-11-58 PMC2924846

[45] Vaananen M, Pietila K, Airaksinen M Self-medication with antibiotics: does it really happen in Europe? Health Policy 2006; 77: 166–71. 16095749 10.1016/j.healthpol.2005.07.001

[46] Muscat M, Monnet D, Klemmensen T, Grigoryan L, Jensen M, Anderson M, et al Patterns of antibiotic use in the community in Denmark. Scand J Infect Dis 2006; 38: 597–603. 16857602 10.1080/00365540600606507

[47] UNICEF State of the worlds’ children. United Nations Children’s Fund, New York, NY 10017, USA, 2007

[48] Fazlul K, Akramul I, Chowdhury A, Johansson E and Diwan V Gender differences in delays in diagnosis and treatment of tuberculosis. Health Policy and Planning. 2005; 22(5): 32934. 10.1093/heapol/czm026 17698889

[49] Thongsuksai P, Chongsuvivatwong V, Suplung H Delay in breast cancer care: a study in Thai women. Med Care 2000; 38(1): 108–14. 10630725 10.1097/00005650-200001000-00012

[50] Noureddine S, Adra M, Arevian M, Dumit N, Shehab D, Abchee A, et al Delay in seeking healthcare for acute coronary syndromes in a Lebanese sample. J Transcult Nurs 2006; 17(4): 341–8. 16946116 10.1177/1043659606291544

[51] Wall L, Karshima J, Kirschner C, Arrowsmith S The obstetric vaginal fistula: characteristics of 899 patients from Jos, Nigeria. Am J Obstet Gynecol 2004; 190: 1011–19. 15118632 10.1016/j.ajog.2004.02.007

[52] Uamai A Review of WHO handbook ‘Monitoring Emergency Obstetric Care’. Paper presented at Training Course in Sexual and Reproductive Health Research, Geneva, 2010 Geneva Foundation for Medical Education and Research; 2010.

